# The Effects of Research & Development Funding on Scientific Productivity: Academic Chemistry, 1990-2009

**DOI:** 10.1371/journal.pone.0138176

**Published:** 2015-09-15

**Authors:** Joshua L. Rosenbloom, Donna K. Ginther, Ted Juhl, Joseph A. Heppert

**Affiliations:** 1 Department of Economics, Iowa State University, Ames, Iowa, United States of America; 2 National Bureau of Economic Research, Cambridge, Massachusetts, United States of America; 3 Department of Economics, University of Kansas, Lawrence, Kansas, United States of America; 4 Department of Economics, University of Kansas, Lawrence, Kansas, United States of America; 5 Department of Chemistry, University of Kansas, Lawrence, Kansas, United States of America; Northwestern University, UNITED STATES

## Abstract

This article examines the relationship between Research & Development (R&D) funding and the production of knowledge by academic chemists. Using articles published, either raw counts or adjusted for quality, we find a strong, positive causal effect of funding on knowledge production. This effect is similar across subsets of universities, suggesting a relatively efficient allocation of R&D funds. Finally, we document a rapid acceleration in the rate at which chemical knowledge was produced in the late 1990s and early 2000s relative to the financial and human resources devoted to its production.

## Introduction

In 2011 the federal government supported nearly $40 billion dollars of university research and development (R&D) activities. Other sources of funding–including industry, state government and the universities themselves–provided over $22 billion in support for university R&D. It is natural to wonder what these investments produce or, put slightly differently, what is the return on this investment? A recent National Science and Technology Council [1] report, for example, observes, “The pragmatic reality facing Federal agencies is that the resources available for investing in research are limited” and argues that there is a need for more systematic, quantitative models relating funding inputs to a variety of significant scientific outputs.

In large part, public investments in R&D are motivated by the conviction that advances in scientific understanding will contribute to the nation’s economic growth. Yet, we still have relatively limited knowledge about the relationship between R&D funding, the production of scientific knowledge, and the effects of this knowledge on socioeconomic outcomes. The first step in linking federal R&D funding to economic growth is to identify whether funding affects knowledge production. Thus, our study examines the effect of federal and non-federal R&D funding for chemistry research on knowledge production in these fields, measured by publications and citations. While it is obvious that funding leads to additional research, our estimation methods account for the effect of funding at the margin: how does an incremental change in research funding contribute to knowledge production at the margin? After controlling for the endogeneity of research funding, we find that that federal and non-federal chemistry research funding increases chemistry publications and citations.

We focus on academic chemistry and chemical engineering in this study for several reasons. To begin with, basic and applied chemistry account for a substantial share—slightly more than 4%—of all federal R&D expenditures. Moreover, the chemical sciences are large, well-established and widely represented across the spectrum of U.S. universities, and they include a breadth of research topics from basic science to highly applied topics. As such, they offer an excellent opportunity to explore the impact of federal and other sources of funding on scientific productivity. Finally, discoveries in chemistry are more likely to result in patents and other forms of commercialization. Thus identifying the effect of federal R&D funding on knowledge production is the first step in linking federal R&D expenditures to product innovations resulting in economic growth and development.

This article contributes to a greater understanding of these relationships through an examination of the relationship between R&D funding and the knowledge outputs produced by academic chemists receiving this funding over the 20 years from 1990 to 2009. Measuring knowledge outputs either by the raw number of articles published or adjusting for the quality of publications by weighting each by the number of citations it received, we document a strong, positive causal effect of funding on knowledge production. At the same time, our analysis also reveals that there was a rapid acceleration in the production of chemical science knowledge in the late 1990s and early 2000s relative to the quantities of both financial and human resources devoted to its production. In contrast to earlier research, which found that numbers of publications grew at roughly the same rate as funding inputs in the 1980s, we find that the number of articles produced grew much more quickly than either financial or human inputs into knowledge production after 1990. Put somewhat differently, we show that the cost per article written fell substantially in these years. Moreover, this trend toward declining costs occurred across a broad spectrum of institutions of higher education.

We begin, in the next section, with a discussion of previous research on the relationship between funding and knowledge production. Next we discuss our data sources and provide an overview of sample characteristics. We then examine aggregate characteristics before turning to an econometric analysis of the relationship between knowledge production and R&D funding.

### The Relationship between Research Funding and Knowledge Production

There is no question that at the aggregate level federal and non-federal research dollars help to support the nation's research enterprise. However, important questions remain about how variations in the level of overall funding from current levels as well as changing the distribution of funding across researchers and institutions at the margin affects knowledge production. Answers to these questions are of considerable relevance to policy-makers seeking to determine the appropriate level of science funding and how to allocate it.

We are not the first to investigate the relationship between R&D funding and knowledge production, but prior research on this topic is rather limited. Previous work has taken a number of different approaches to this problem, reflecting different conceptions of the appropriate unit of measurement. One approach, exemplified by a recent study by Jacob and Lefgren [2], looks at the impact of receiving a grant on the productivity of an individual researcher. Utilizing administrative data from the National Institutes of Health (NIH) on all training and standard grant proposals submitted between 1980 and 2000, Jacob and Lefgren estimate the effects of being awarded a grant versus not receiving the grant on a scholar’s subsequent publication. To control for project quality they estimate the causal effects of a successful grant application through a regression discontinuity design that exploits the fact that funded and unfunded proposals with similar priority scores are comparable in quality. Although they do find a positive effect of funding on subsequent publication, the size of this effect appears quite modest, resulting in approximately one additional publication in the subsequent 5 years. Although it remains unpublished, work by Arora and Gambardella [3] adopts a similar methodology in examining the effects of grants from the National Science Foundation’s (NSF) Economics program. Like Jacob and Lefgren [2], they find a relatively modest effect of funding on subsequent productivity, although this effect varies by researcher seniority.

These results are intriguing, but as Jacob and Lefgren acknowledge, they are subject to important limitations. Most significantly, it is not possible, given their empirical set up, to determine whether investigators placed in the control group because they did not receive funding in a particular funding round may have been successful in a later resubmission or were able to obtain resources for their research from another source. To the extent that the control-group includes investigators who subsequently obtained funding, the estimated effects of receiving a grant will be biased downward.

Another factor that will produce a downward bias in estimates at the individual level is the likely impact of leakages and spillovers on the effects of funding. Research support to individual academic researchers includes the direct costs of the research and sizeable payments to their university employers in the form of “overhead” or indirect costs (Facilities & Administration) in order to support the broader scientific enterprise. These resources presumably support a broader set of researchers and research infrastructure. Further, the activity and resources of funded investigators may indirectly encourage research output by unfunded colleagues by providing a foundation of support for these investigators and supporting the institutional resources that benefit the research activities of their graduate students and postdoctoral researchers.

To better capture the effect of these spillovers, several researchers have examined how R&D funding affects outputs at the level of universities and the university system as a whole. Blume-Kohout, Kumar and Sood [4] investigated whether federal R&D life science funding was a complement or a substitute for non-federal R&D funding. They found that federal and non-federal funding were complements. Adams and Griliches [5] examined the relationship between R&D funding and research publications at 109 universities in the period 1981–1993 by analyzing aggregate spending and publications in 8 broad disciplinary categories. Relying on an informal analysis of graphs showing the growth of R&D expenditures and publications or citations in each discipline, they concluded that at the aggregate, university-system level, funding and scholarly outputs grew roughly in parallel with one another in most of the disciplines they considered. They then used panel regressions for a subset of universities with complete data to estimate the elasticity of publications or citations relative to funding. In contrast to the elasticity near one suggested by the aggregate data, they found cross-section elasticities in the range of about 0.4, up to a high of 0.9. They suggested that this discrepancy might be attributable to the leakages and spillovers alluded to earlier.

More recently, Payne and Siow [6] have examined the connections between federal R&D funding and research output. Their analysis is more aggregated than Adams and Griliches, however, reporting a single aggregate estimate across all disciplines of the effect of federal R&D funds on research output using a panel of 57 universities for the period 1981–1998. Payne and Siow recognized that because R&D funding is not allocated randomly across institutions, Ordinary Least Squares (OLS) regression results cannot be interpreted as reflecting the causal effect of funding on output. To resolve this latter problem, they proposed an instrumental variables approach that relies on the effect of university alumni representation on key congressional appropriations committees. Using this instrument they found that an additional $1 million in federal R&D funding resulted in an increase of approximately 10 publications. Further, they concluded that there was no relationship between the level of funding and the number of citations per article. Payne and Siow’s instrument lacks power, however, and the impact of congressional representation on the award of merit-based scientific R&D awards might be questioned a priori.

Like Payne and Siow[6], Whalley and Hicks [7] examined the relationship between university research expenditures and knowledge production across the full spectrum of different disciplines. As in the other studies mentioned earlier Whalley and Hicks measured knowledge production using publications, citations and patents for a panel of 96 research universities between 1985–1996, but they controlled for the endogeneity of funding by using changes in university endowment values as a source of exogenous variation in research expenditures. They found that increases in research expenditures increased the number of publications but had no causal impact on the average citations per paper or the number of patents.

Investigating the relationship between R&D funding and energy research, Popp [8] has used a very similar approach to these other studies, but rather than focusing on variation across universities, he assembled panel data on publications and R&D funding for several different alternative energy technologies across over a 20 year period in 14 different countries. His estimates indicate that an additional $1 million of government R&D funding leads to between 1 and 2 additional publications, but that the lags between funding and publications can be as long as 10 years. Popp also examined scale effects, concluding that there is no evidence of diminishing returns on the level of publications, but that increased funding may result in lower quality publications.

Most of the previous literature described above is dated, examining the impact of research funding on knowledge production through the mid-1990s. Given the changes in technology, including the personal computer and Internet revolutions, the relationship between federal R&D funding and knowledge production has likely changed. These studies also miss important changes in federal funding priorities such as the NIH budget doubling (1998–2003) [8] as well as substantial increases in the NSF budget during the same period. From 1998 to 2004, NSF R&D obligations increased from $2.3 billion to $3.8 billion, measured in current dollars. While not as impressive as the increase in NIH budgets, this represents a significant infusion of funding and a departure from the relatively stable funding in the years before and after these dates [9]. Furthermore, Payne and Siow [6] and Whalley and Hicks [7] combine R&D funding and aggregate publication and citation counts across all disciplines at the university level. This high level of aggregation would likely “average out” important shifts in federal funding priorities in previous decades (such as the NIH Doubling).

Like these previous studies, we exploit variations in funding levels over time across a panel of universities. Focusing on the behavior of the university-system broadly and on the output of individual universities is more likely to capture the ways in which research funding supports the broader scientific system. Yet unlike previous studies (with the exception of Adams and Griliches), we believe it is important to look at these effects at the level of individual disciplines because of differences across the disciplines in knowledge production, citation patterns, and the uses of funding. This approach is supported by the differences in the funding-output relationship documented by Adams and Griliches in their earlier study [5].

## Data

We have collected data on the levels of research funding along with publication and citation data in chemistry and chemical engineering (for brevity we will refer to these combined fields as chemistry) for a sample of 147 universities over twenty years, from 1990 through 2009. We provide a brief description of these data here, and additional details about sources and methods of merging the different data sources are provided in a data appendix. Although there are likely to be differences in research practices and professional culture between chemistry and chemical engineering, it is not practical to cleanly distinguish between these two fields in the allocation of R&D funding or publications. To illustrate this point, of the 150 institutions we initially examined, there were 102 that had chemical engineering departments that reported faculty numbers in the American Chemical Society directory and 48 that did not. However, only 22 institutions reported zero amounts of federally funded R&D expenditures for chemical engineering research in every year, and there are no institutions for which Web of Science recorded zero chemical engineering publications in all years. The mismatch in classification across the different sources used in our analysis suggests that attempting to analyze these fields separately would likely cause more problems than it solves. Numerically, chemistry accounts for the bulk of publications, R&D expenditures and faculty, and results that are restricted to chemistry look similar to those we report below. Results for chemical engineering resemble in sign and magnitude the results for chemistry, but effects are not significant, possibly because of the smaller sample and likely discrepancies in classification across the different data sources.

Our sample was selected to include those universities that accounted for the bulk of sponsored Research & Development expenditures in Chemistry. To identify our sample institutions, we aggregated federally funded chemistry R&D expenditures (in constant dollars) for the 20 years from 1990 through 2009 and ranked them on the basis of this total. Initially we selected the top 150 institutions, but later concluded it was necessary to drop three of these for which the data were incomplete or appeared inconsistent. The three institutions dropped from our sample included two academic medical centers: the University of California San Francisco and the University of Texas M.D. Anderson Cancer Center—which reported no chemistry faculty, graduate students or postdocs for much of the study period—and the Oregon Institute of Science and Technology, which disappears from the data after 2001. After identifying the sample, we collected additional data on inputs in the knowledge production process, including degrees awarded, graduate student enrollment, postdoctoral researchers employed, and faculty from publicly available sources, and merged these with the R&D expenditure data.

Data on publications and citations were provided by Thomson Reuters, Research Analytics group from the Web of Science citation data base. The Web of Science data are drawn from a selective set of top tier international and regional journals across all areas of the sciences and social sciences. Subject matter experts at Thomson Reuters are responsible both for selecting these journals based on objective and subjective measures of impact. Within this universe of publications, the Thomson Reuters subject matter experts then classify journals into various subject area classifications. Counts of publications (and citations) within each subject area represent the total of all items appearing in journals within a subject classification. We worked closely with Thomson Reuters to identify and match publications in the chemistry and chemical engineering subject categories to our sample institutions on the basis of author affiliations and address information.

A list of each of the institutions included in our sample is provided in S1 Table, which also summarizes a number of key dimensions of real R&D expenditures and outputs. As this table illustrates, our sample exhibits a considerable degree of institutional variation. At the top of the list are institutions such as the Massachusetts Institute of Technology (MIT), Cal Tech, and the University of California Berkeley, with average annual R&D expenditures for chemistry in the $20-$30 million range, employing more than 100 postdoctoral researchers, training more than 50 doctoral recipients per year and producing many hundreds of publications. At the bottom of our list are institutions such as Cleveland State University or North Carolina Agricultural & Technical State University, with total R&D expenditures of little more than $1 million per year, with few or no postdoctoral researchers and doctoral recipients, and producing just tens of publications.

**Fig 1:**
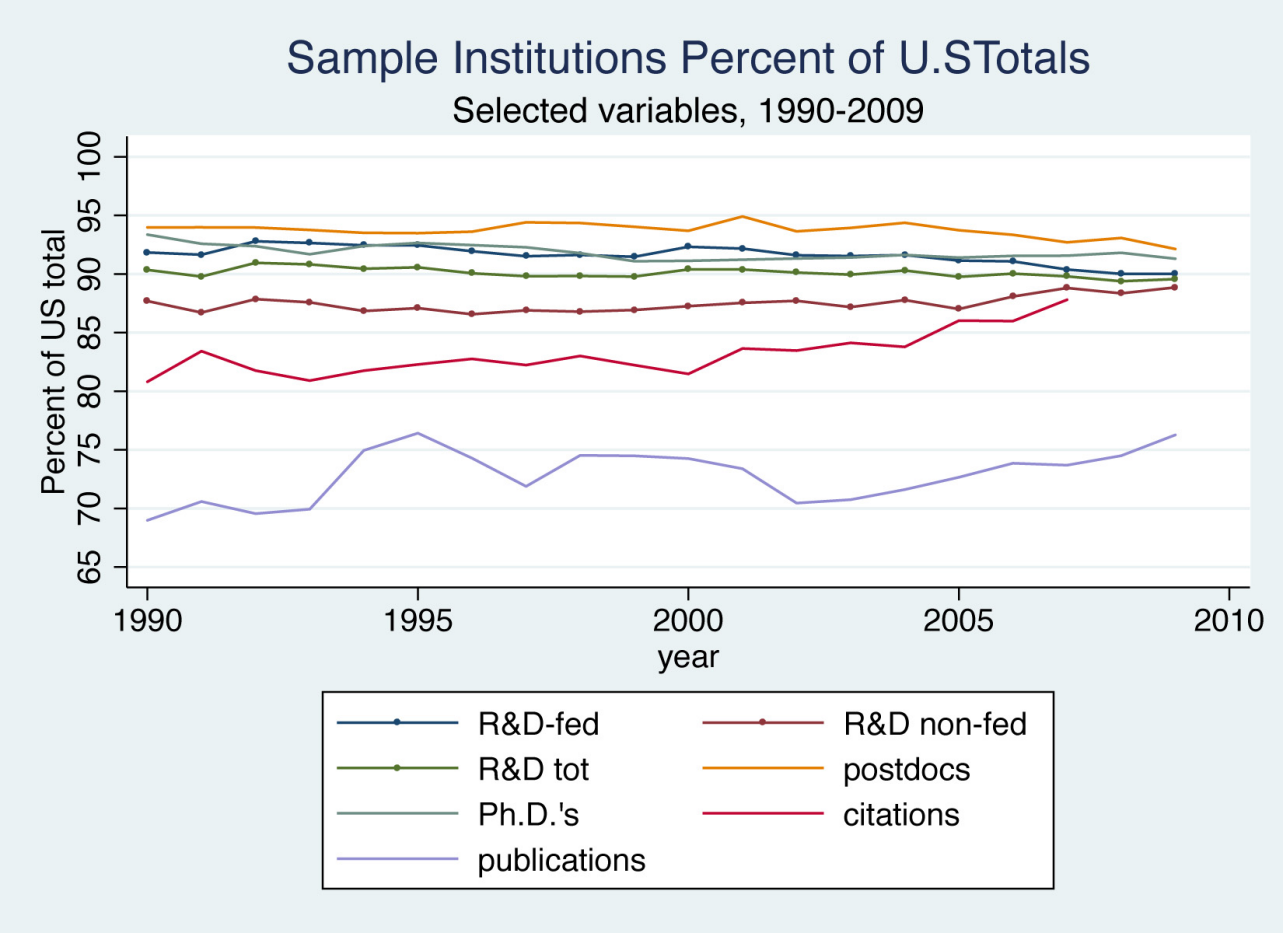
Sample Institutions, Percent of U.S. Totals, Selected Variables, 1990–2009.

Although the 147 institutions we study do not comprise the full extent of academic research in chemistry, they account for the vast majority of the measurable research and training activities in the United States. As Fig 1 illustrates, their shares of total U.S. research expenditures, Ph.D.s awarded and postdoctoral researchers hovered around 90–95 percent, although their share of non-federally funded research expenditures was somewhat lower at about 87 percent. In comparison to R&D expenditures and graduate education, the number of publications is relatively less concentrated. Our sample institutions produced between 70 and 75 percent of chemistry publications in most years. These publications, however, received 80 to 85 percent of citations to U.S. publications over this period, suggesting that researchers affiliated with these institutions produced a greater proportion of the more important publications.

It should be noted that while the institutions in our sample accounted for a stable or slightly rising share of U.S. publications and citations, the United States share of total global publications in chemistry appears to have been declining somewhat over time. Based on data provided by Thomson Reuters from their Web of Science database, from 1990 through the early 2000s, U.S. publications accounted for about 30 percent of all chemistry publications, but after 2003, this figure began to drop, falling closer to 25 percent by 2009. U.S. publications do, however, receive a greater proportion of total citations, suggesting that they remain more important in global chemistry than the raw publication count would indicate. This share was also declining, however, over the last decade or so.

Table 1 provides additional details about the characteristics of both the full sample and several important subsets of universities, reporting average annual values of key variables for the periods 1990–1999 and 2000–2009. Across all universities, average annual chemistry R&D expenditures increased from an average of about $8 million in the 1990s to almost $11 million in the 2000s. Federal sources supported just under two-thirds of R&D spending in both periods and grew at roughly the same rate as overall R&D spending. In contrast to this growth in expenditures, average numbers of graduate students enrolled, Ph.D.s awarded and employment of postdoctoral researchers held relatively steady across the two decades. On the other hand, the average number of publications produced and the number of citations to those publications both nearly doubled. The growth in the number of publications was due in part to a substantial increase in the number of chemistry journals included in the Web of Science database, which increased from 244 in 1990 to 568 by 2009. However, even within a fixed set of journals that were included in the Web of Science in 1990, there was an approximately 80 percent increase in the number of chemistry publications.

**Table 1:** Annual Average Values of Key Variables, by Decade and University Characteristics.

	Full	Research Status		Control	
	Sample	Research 1	Other	Private	Public
	**1990–1999**				
Federally Funded R&D (thousands)	$ 5,203	$ 7,190	$ 2,559	$ 6,010	$ 4,823
Federally Funded Equipment Expenditures	$ 515	$ 704	$ 263	$ 582	$ 483
Federally Funded Non-Equipment Expenditures	$ 4,688	$ 6,486	$ 2,297	$ 5,427	$ 4,340
Non-Federally Funded R&D (thousands)	$ 2,821	$ 3,596	$ 1,791	$ 2,024	$ 3,196
Non-Federally Funded Equipment Expenditures	$ 253	$ 311	$ 177	$ 184	$ 286
Non-Federally Funded Non-Equipment Expenditures	$ 2,568	$ 3,285	$ 1,614	$ 1,840	$ 2,910
Total R&D Expenditures	$ 8,024	$ 10,786	$ 4,350	$ 8,034	$ 8,019
Percent R&D Federally Funded	64.6%	66.1%	62.5%	72.0%	61.1%
Full Time Faculty	33	40	24	28	35
Graduate Students Enrolled	150	190	96	126	161
Ph.D.s awarded	18	25	8	16	19
Postdoctoral Researchers	27	39	11	29	26
Number of publications	120	174	48	113	123
Total 3-year citations	832	1,295	211	1,041	734
	**2000–2009**				
Federally Funded R&D (thousands)	$ 6,954	$ 9,491	$ 3,571	$ 7,048	$ 6,909
Federally Funded Equipment Expenditures	$ 524	$ 687	$ 307	$ 483	$ 543
Federally Funded Non-Equipment Expenditures	$ 6,430	$ 8,804	$ 3,264	$ 6,565	$ 6,366
Non-Federally Funded R&D (thousands)	$ 3,902	$ 5,196	$ 2,177	$ 2,370	$ 4,622
Non-Federally Funded Equipment Expenditures	$ 344	$ 450	$ 204	$ 192	$ 416
Non-Federally Funded Non-Equipment Expenditures	$ 3,558	$ 4,746	$ 1,974	$ 2,179	$ 4,206
Total R&D Expenditures	$ 10,856	$ 14,687	$ 5,749	$ 9,418	$ 11,532
Percent R&D Federally Funded	66.2%	66.2%	66.2%	73.8%	62.6%
Full Time Faculty	34	41	26	29	36
Graduate Students Enrolled	158	205	95	135	168
Ph.D.s awarded	18	26	8	16	19
Postdoctoral Researchers	31	44	13	34	29
Number of publications	209	297	92	200	213
Total 3-year citations	1,650	2,465	562	1,857	1,552
Number of institutions	147	84	63	47	100

Other scholars have documented a growth in the size of research teams and author lists. Since we count publications at the university level, an increase in the number of authors will not increase our article count so long as all authors are affiliated with the same university. When author affiliations include more than one university, we count the article twice, but there was only a modest increase in the number of articles of this type, from 13 percent of all articles in 1990 to 22 percent in 2009. Although the double counting of articles with multiple institutional affiliations produces a small upward bias in total publication counts, this effect is quite small. For the institutions in our sample, the growth of articles with a single institutional affiliation increased by a factor of 2.4 between 1990 and 2009. Eliminating double counting would thus slightly moderate the growth rates we find, but would not substantively alter our results.

We categorized universities based on their Carnegie ranking and public or private status. Comparing across categories, important differences emerge in Table 1. As we might expect, all of the indicators of both inputs and outputs of the chemical sciences are much larger at those universities in the Carnegie Research I classification than at the other, non-Research I institutions in our sample. The Research I universities accounted, on average, for about 2.5 times the research expenditures and produced nearly three times as many doctorates as the non-Research I universities. They also employed more than three times as many postdoctoral researchers and produced more than three times as many publications. The imbalance in citations to publications was even more striking: for 2000–2009, publications produced by the Research I institutions received almost 4.5 times as many citations as those produced by the non-Research I group, down from a ratio of more than 6 in the 1990s.

Although average research expenditures at public and private universities were similar in the 1990s, their composition was somewhat different, with non-federal funding making up almost 40 percent of total expenditures at public universities, compared to less than 30 percent at the private universities. These differences persisted over time, but funding received by the public universities grew more quickly than did funding at the private universities. Average numbers of graduate students enrolled were also higher at the public universities, but the numbers of doctorates awarded and postdoctoral researchers were comparable across the two groups. The average number of publications produced by public and private universities was quite similar, but private university publications received, on average, more citations than did those produced by the public universities. If citations provide a measure of the significance of publications, this result suggests that private universities were, on average, producing research of somewhat higher quality than their public counterparts.

### Aggregate Trends in Chemistry Research and Funding

**Fig 2:**
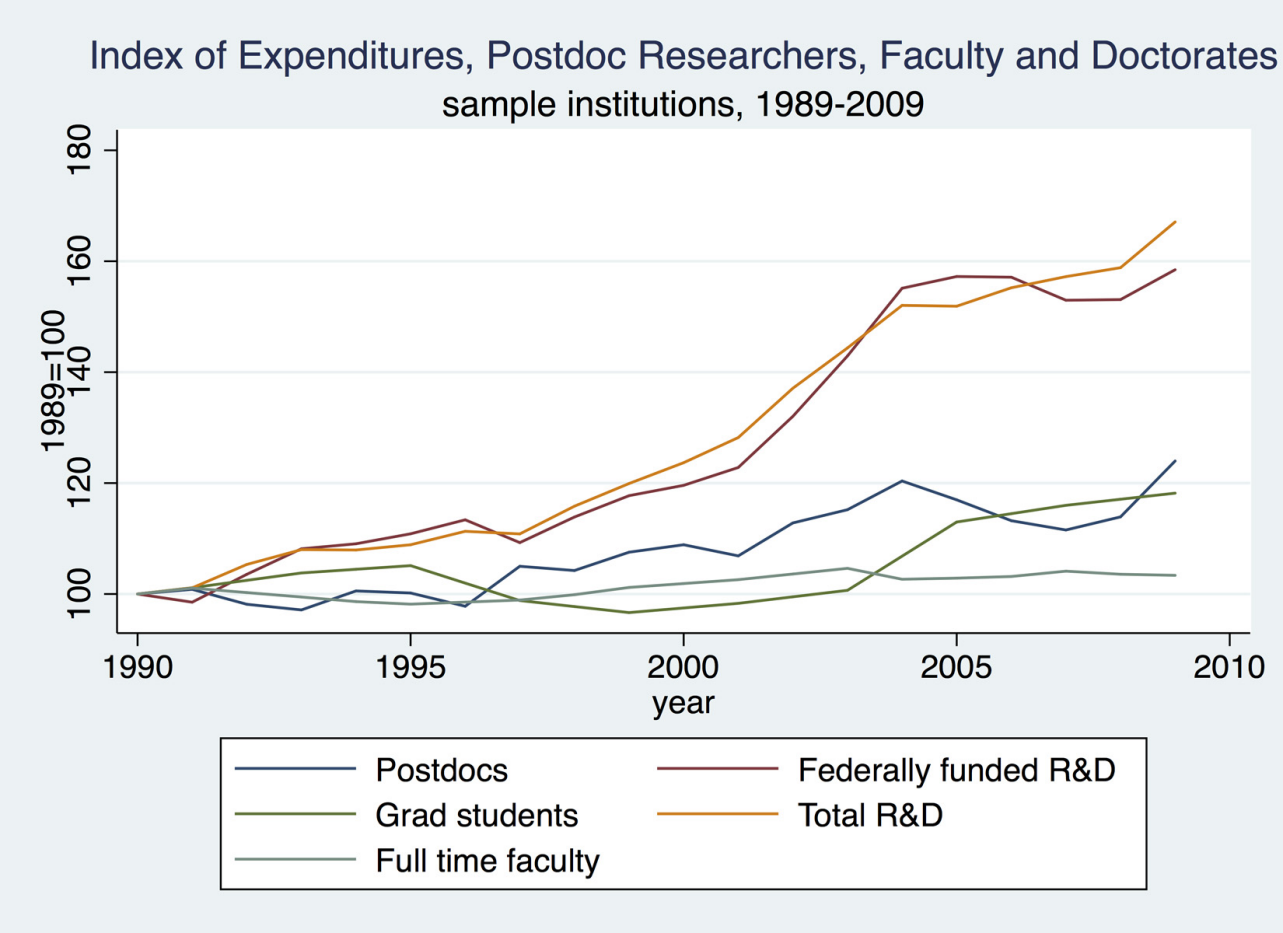
Expenditures, Postdoc Researchers, Faculty and Doctorates, Sample Institutions, 1989–2009.

Before exploring the causal effect of funding on knowledge production, we consider some of the aggregate characteristics of research funding and scholarly outputs of academic chemistry over the last two decades. We begin with the growth of funding and personnel. Fig 2 compares the growth of R&D expenditures (federally financed and total) with the number of doctorates awarded and numbers of full-time faculty and postdoctoral researchers employed. To facilitate comparison, each series is graphed as an index (set equal to 100 in 1990). Over most of the period, federal and non-federal funding grew at comparable rates, but since 2006, federal funding has stabilized, while funding from non-federal sources has continued to increase.

In comparison to the nearly 70 percent increase in total R&D funding, faculty numbers were essentially flat over this 20 year period, while the number of postdoctoral researchers increased only modestly. The number of postdoctoral researchers did increase somewhat after 1996 and had grown by about 20 percent by the end of our study period in 2009. The number of doctorates awarded fluctuated with no clear trend until the early 2000s when it also began to rise slowly. Again, however, this increase was modest compared to the increase in R&D expenditures.

**Fig 3:**
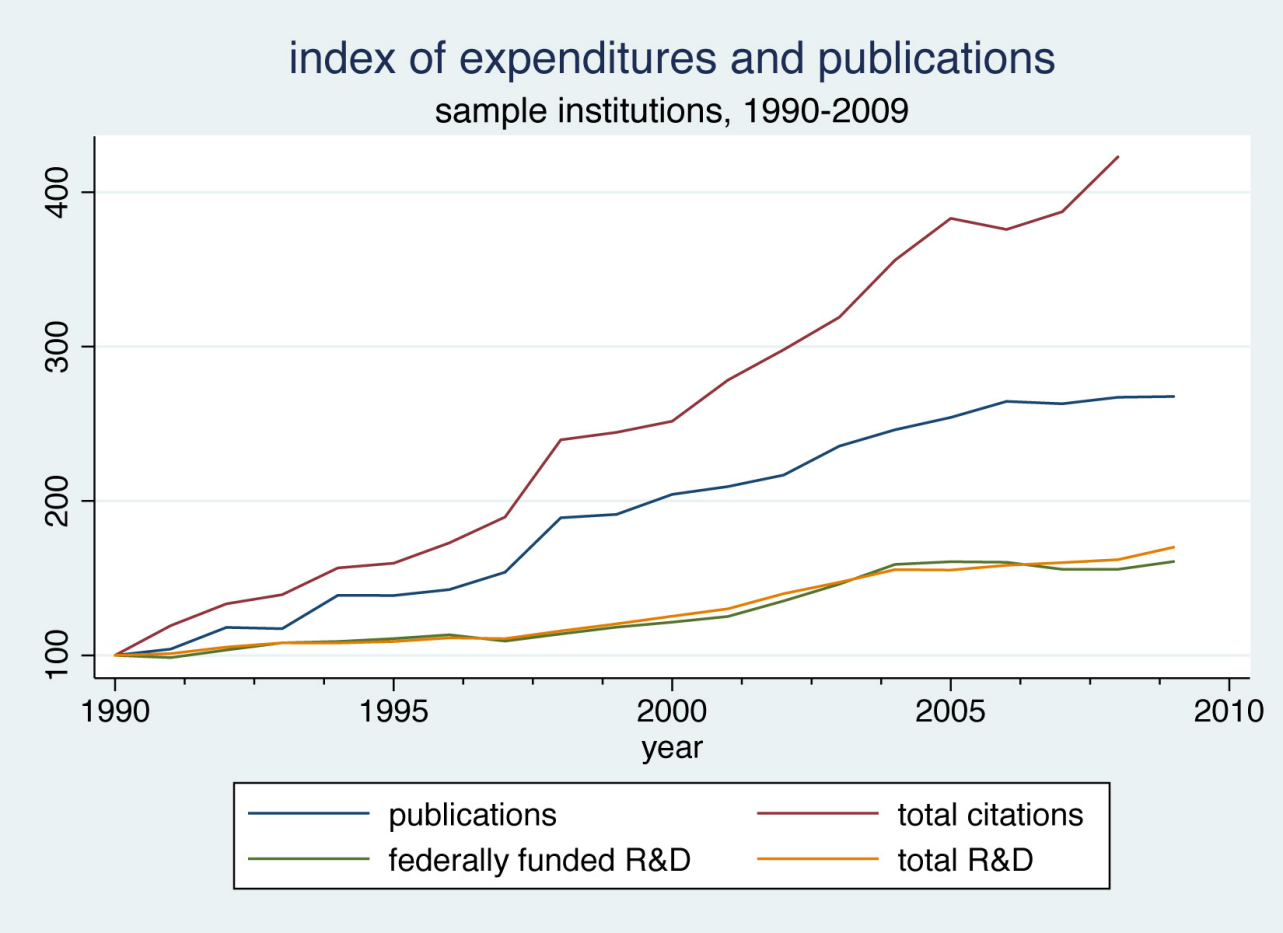
Index of Expenditures and Publications, Sample Institutions, 1990–2009.

In Fig 3 we shift the focus to measures of research output, graphing the growth in numbers of publications and citations for our sample institutions. As in Fig 2, we have plotted each series as an index with 1990 set equal to 100, and we have included the indexes for federally-funded and total R&D expenditures for comparison. It is apparent that academic chemistry publications and citations increased much more rapidly than did funding in this period. The index of publication numbers increases consistently and reaches a value of 268 by 2009, nearly a threefold increase. Meanwhile, the index of citations to these publications grows even more quickly, achieving a value of 422 in 2008 (the last year for which we can calculate a three-year citation count).

The relationships in Fig 3 can be compared with those reported by Adams and Griliches [5]. They found that between 1981 and 1993, publications and R&D expenditures in chemistry increased at very nearly the same rate; both increasing approximately 50 percent. Thus there appears to be a change in the knowledge production function at the aggregate level for the chemical sciences in the more recent period.

**Fig 4:**
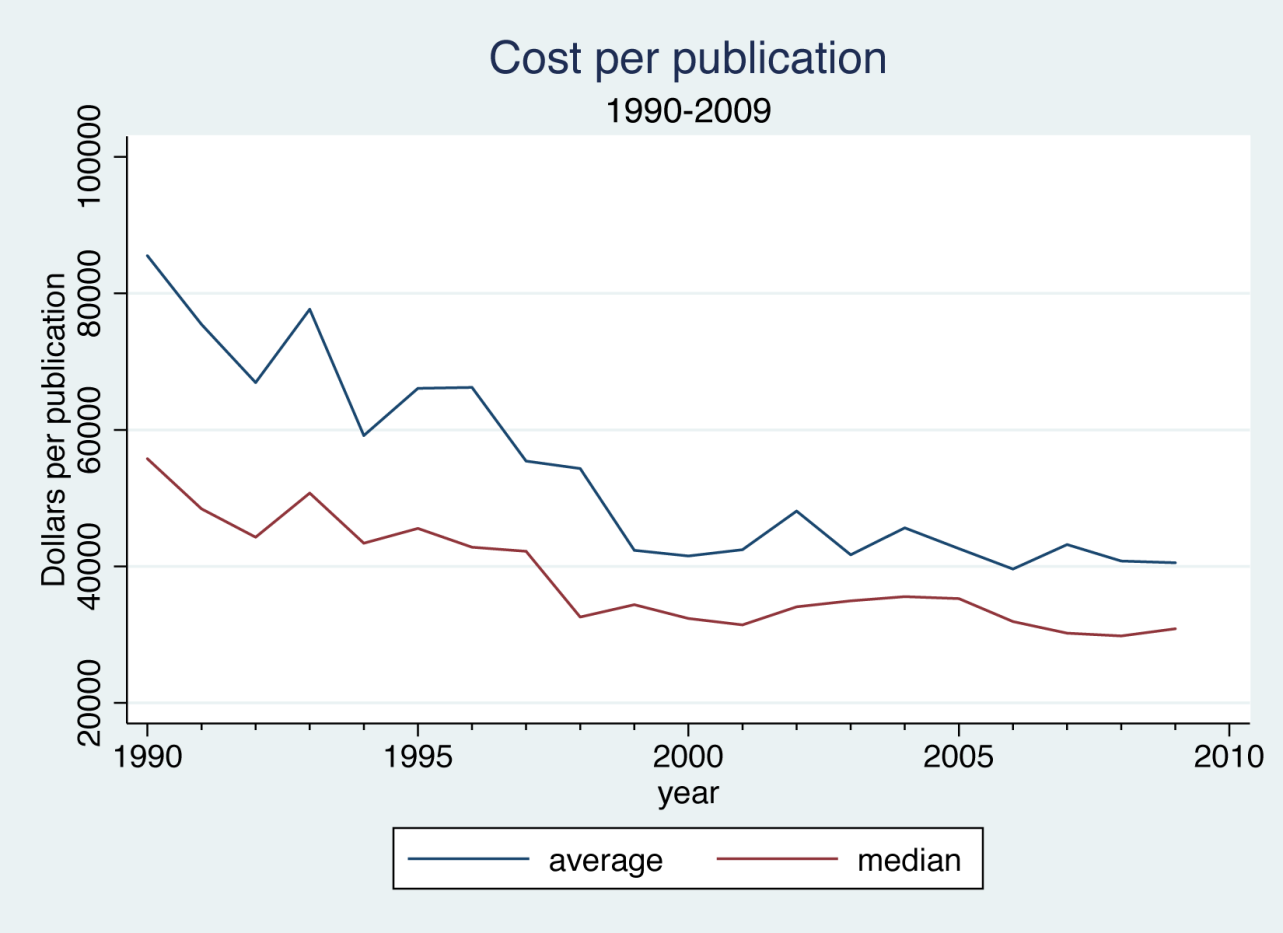
Cost per Publication, 1990–2009.

**Fig 5:**
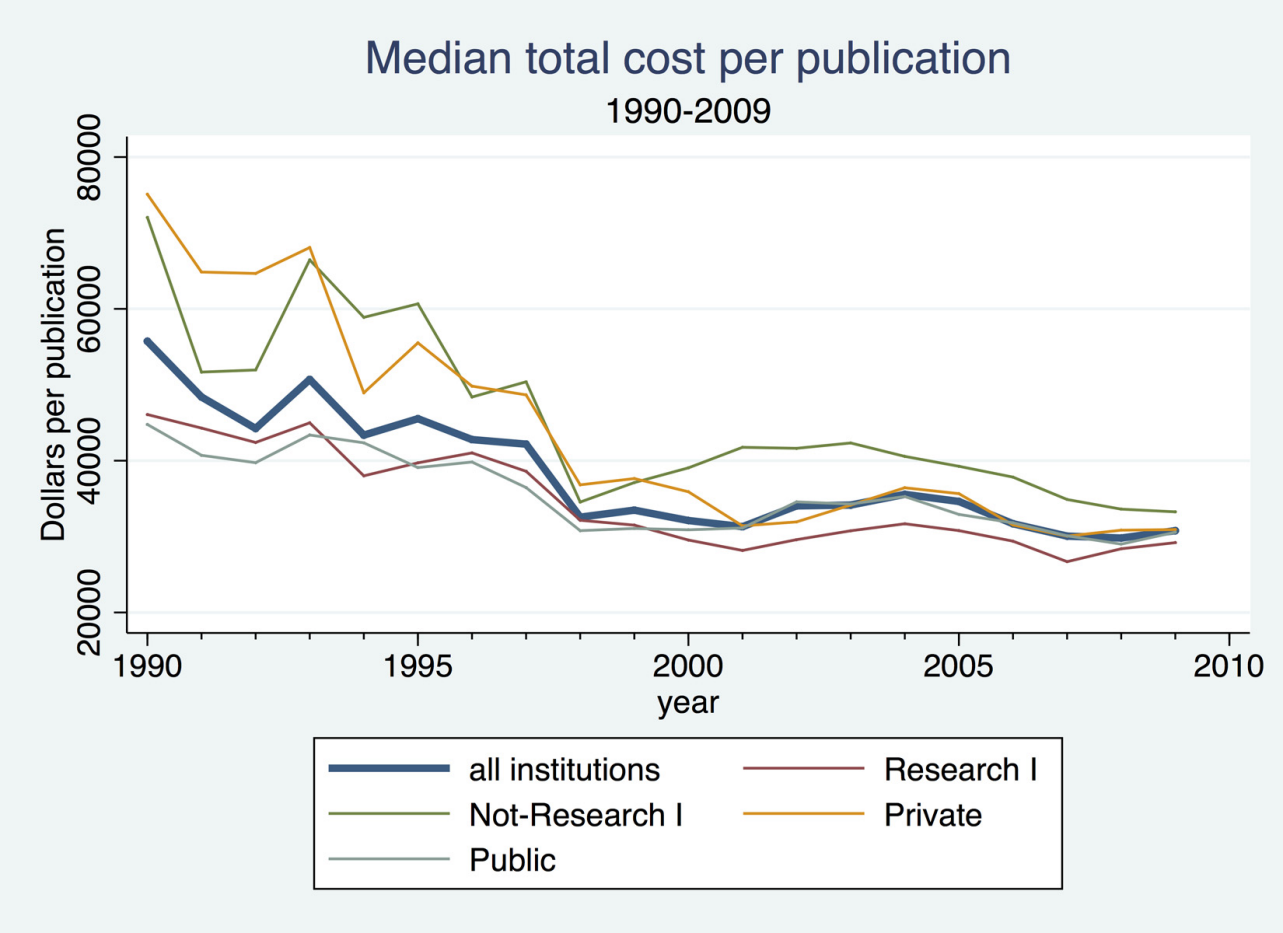
Median Total Cost per Publication, 1990–2009.

The data on outputs and expenditures shown in Fig 3 make it clear that at the aggregate level the cost per publication was declining over time. In Figs 4 and 5, we use the university-level data to look more closely at the relationship between inputs and outputs over time. Dividing total chemistry R&D expenditures at each institution by the number of chemistry publications attributed to that institution yields an average cost per publication. Fig 4 plots the mean and median of the resulting distribution of average cost per publication in each year. Both measures fell appreciably between 1990 and 1998 then leveled off. The median fell from just under $60,000 per article to around $30,000 per article. The divergence between the mean and the median reflects the impact of a few extremely high-cost universities, and the convergence of these two measures over time suggests that costs per publication were becoming somewhat less skewed over time.

In Fig 5 we compare the median cost of publications across subsets of universities defined by their research intensity and control (i.e., public or private). In each group the time trends are similar, and the differences in levels appear reasonable: costs are lowest at the more research-intensive universities and the public universities. Perhaps of greater interest, however, is the apparent convergence of costs across the different groups over time. Median costs fell much more sharply at less research-intensive and private universities, producing a considerable convergence of average costs by the late 1990s. Costs per publication appear to have risen again at the non-Research I universities during the early 2000s, but then began to fall after the early 2000s.

### Knowledge Production and Research Funding at the University Level

As a rule, universities produce new scientific knowledge by combining labor (faculty, postdoctoral researchers, graduate students), with capital (buildings, laboratories, infrastructure, and specialized equipment), and other purchased inputs. While faculty salaries are supported primarily from institutional sources, support for postdoctoral researchers, graduate students, and most purchased inputs comes primarily from externally-sponsored research funding from either federal or non-federal sources. Structures and other long-lived capital equipment used in knowledge production may be treated as an institution-specific fixed effect. Formally, we can express this relationship in the following knowledge production function:

$$y_{it}=f\left(A_{t},L_{it-1},R_{it-1}^{f},R_{it-1}^{n},\alpha_{i}\right)$$

Where i indexes institutions, t denotes time periods, L is faculty labor input, R^f^ and R^n^ denote, respectively, federal and non-federal funding, α is an institution specific effect, and A is included to capture technical changes (and other factors) that are common across all universities at a point in time. The time subscripts on the input variables are all denoted as t-1 to reflect the fact that there is typically a time lag between the input of resources and the publication of research results. For a somewhat different approach to modeling knowledge production, which accounts for the joint production of publications and graduate student training, see Apon et al. [10], who use a stochastic frontier production function model. Hare and Wyatt [11] and de Groot [12] provide further discussion of approaches to modeling knowledge production functions.

Theory offers no guidance about the specific functional form that the knowledge production function takes. In what follows, we estimate a simple linear approximation to this function:

$$y_{it}=\alpha_{i}+A_{t}+\gamma_{f}R_{it-1}^{f}+\gamma_{n}R_{it-1}^{n}+\delta L_{it-1}+\varepsilon_{it}$$

where ε is a stochastic error term that is assumed to be independently distributed over time for each institution. In our estimation we use faculty numbers and research funding from the prior year. Initially we had hoped to directly estimate the lag-structure of the relationship by including multiple years of lagged inputs on the right hand side of the equation. It became apparent, however, that these variables were highly collinear, making it impossible to precisely estimate their separate effects. When it became clear that we could not use this strategy, we experimented with a number of ad hoc specifications, including the average levels of inputs over the previous five years, a declining sum of the previous five years’ inputs, and an inverted V weighting structure. All produced essentially the same results as a one-period lag. It is certainly true that some publications are the result of efforts expended much longer than one year ago, but research expenditures and faculty numbers are highly serially correlated and experimentation with a number of different approaches to approximating the production lags all yield quite similar results.

We measure knowledge outputs in two ways. The first is a simple count of the number of publications in year t that are attributed to the institution based on the affiliations recorded for each author. The vast majority of articles in our data have authors with only one institutional affiliation. However, when the authors of an article have affiliations with more than one institution, we credit each institution with the publication. Because there was a small increase in the number of multi-affiliation articles over time, this approach introduces a small upward bias into the time trend, but it can account at most for an increase of perhaps 5–10% in the total number of articles over the period.

The second measure of knowledge output counts the number of citations that articles published in year t receive during a three-year window beginning in the publication year. We also considered extending the citation window to 5-years, but the results of the two approaches are quite similar. To the extent that citations may be interpreted as a reflection of the significance of each publication, we can treat the citation count as a relative quality-adjusted measure of knowledge production. We say “relative” quality because, as noted earlier, the average number of citations per article increased substantially between 1990 and 2009, which we interpret as a change in citation practices that may reflect the greater visibility of publications as a result of the movement toward electronic publication and distribution. Thus, the absolute number of citations is unlikely to reflect the absolute quality of an article, but it will still provide evidence about the relative quality of articles published at the same time.

#### OLS Fixed Effect Results

We begin by reporting OLS estimates of equation (2). Table 2 reports coefficient estimates for the key explanatory variables in equation (2) for three dependent variables: the number of publications, the number of citations received by those publications, and the average number of citations per publication. For both total publications and citations, the coefficients on both lagged federal and non-federal funding are positive, statistically and economically significant, and of approximately the same size. Since the funding variable is measured in $1000s, they imply that an additional $1 million in funding results in between 6 and 7 more publications in the following year, and 60 to 70 more citations to those publications. This implies a marginal cost per publication in the range of $150,000. For comparison it is worth noting that in the previous section we found that the average cost per publication fell from around $80,000 in 1990 to about $40,000 by the early 2000s. Given the relatively small changes in the number of full-time faculty in the years being considered, it is not surprising that the coefficient on this variable is consistently small and statistically insignificant. Any effects of faculty size must be absorbed in the university fixed effects or in variations in the scale of funding. Overall, the model does a reasonably good job of accounting for the observed patterns of variation in publications and citations, explaining over half of the observed variation in the dependent variables. It should be pointed out that this is not simply a result of cross-sectional variation across institutions, as the R-squared values for within variation are also relatively high, indicating that temporal variations in funding at a university account for a good deal of the temporal variation in research output.

**Table 2:** OLS Panel Regressions Determinants of Publications, Citations, and Average Number of Citations per Publication.

Dependent Variable	Number of Publications		Number of Citations (3-Year Horizon)		Average Number of Citations per Publication	
	Model l	Model ll	Model l	Model ll	Model l	Model ll
L.Federally Funded R&D		0.00609***		0.0645***		0.0000568
		(0.00125)		(0.0177)		(0.0000414)
L.Non-Federally Funded R&D		0.00669***		0.0693**		0.0000606
		(0.00177)		(0.0240)		(0.0000476)
L.Total R&D Funding	0.00634***		0.0665***		0.0000584*	
	(0.00107)		(0.0144)		(0.0000290)	
L.Full Time Faculty	-0.209	-0.213	-1.227	-1.263	-0.00175	-0.00178
	(0.224)	(0.227)	(3.171)	(3.233)	(0.0119)	(0.0119)
Year Effects	Yes	Yes	Yes	Yes	Yes	Yes
Constant	46.61***	47.10***	59.61	63.54	6.926***	6.929***
	(11.87)	(11.86)	(152.8)	(155.0)	(0.472)	(0.477)
Observations	2933	2933	2933	2933	2933	2933
sigma_e	43.34	43.34	577.9	578.0	2.168	2.168
sigma_u	93.34	93.78	935.1	940.2	2.953	2.956
rho	0.823	0.824	0.724	0.726	0.650	0.650
r2_w	0.619	0.619	0.484	0.484	0.241	0.241
r2_b	0.757	0.751	0.624	0.615	0.194	0.188
r2_o	0.581	0.576	0.503	0.498	0.156	0.154

Standard errors in parentheses.

* p<0.05,

** p<0.01,

*** p<0.001.

Notes: All regressions include year and institution fixed effects. Estimated using STATA xtreg procedure with cluster robust standard errors.

The third specification, which examines the relationship between research funding and the average number of citations per publication, allows us to consider whether there is an effect of funding on the quality of publications, independent of their quantity. As the estimates for this dependent variable indicate, none of the included explanatory variables contribute significantly to the average publication quality.

In Table 3 we disaggregate the sample of universities to explore whether the effects of funding on knowledge production vary by control (public vs. private) or research intensity (Carnegie Research I institutions vs. non-Research I institutions). The effects of federal R&D funding are statistically significant across all of the different subsets, and while the point estimates vary somewhat, the differences in these coefficients are not large enough to be significant. In contrast, there is marked variation in the effects of non-federal research funds. For both private universities and non-Research I institutions, non-federal funds appear to have a much smaller effect and one that is not significantly different from zero. For the average number of citations per publication, the effects of funding in the disaggregated regressions remain small in magnitude, however, there is a small, positive, and statistically significant effect on average citations for public universities and for the non-Research I universities.

**Table 3:** OLS Panel Regressions. Determinants of Publications, Citations, and Average Citations per Publication, by University Type.

	Full sample	Private	Public	not Research l	Research l
	**Panel A-Dependent Variable: Number of Publications**				
L.Federally Funded R&D	0.00609***	0.00652***	0.00611***	0.00404*	0.00523***
	(0.00125)	(0.00173)	(0.00171)	(0.00172)	(0.00131)
L.Non-Federally Funded R&D	0.00669***	0.00293	0.00775***	0.00294	0.00536**
	(0.00177)	(0.00347)	(0.00198)	(0.00177)	(0.00201)
L.Full Time Faculty	-0.213	-0.156	-0.282	0.0659	-0.228
	(0.227)	(0.418)	(0.308)	(0.203)	(0.341)
Year Effects	Yes	Yes	Yes	Yes	Yes
Constant	47.10***	41.06	51.43***	17.41*	82.24***
	(11.86)	(23.75)	(14.50)	(7.789)	(18.25)
Observations	2933	940	1993	1256	1677
sigma_e	43.34	44.79	42.60	23.50	47.27
sigma_u	93.78	89.93	95.36	39.37	94.30
rho	0.824	0.801	0.834	0.737	0.799
r2_w	0.619	0.578	0.642	0.572	0.710
r2_b	0.751	0.799	0.718	0.605	0.675
r2_o	0.576	0.602	0.569	0.430	0.539
	**Panel B-Dependent Variable: Number of Citations**				
L.Federally Funded R&D	0.0645***	0.0527	0.0749***	0.0343*	0.0566**
	(0.0177)	(0.0338)	(0.0201)	(0.0131)	(0.0202)
L.Non-Federally Funded R&D	0.0693**	0.0578	0.0760**	0.0289*	0.0607*
	(0.0240)	(0.0434)	(0.0273)	(0.0125)	(0.0284)
L.Full Time Faculty	-1.263	-2.391	-0.853	0.272	-0.427
	(3.233)	(4.238)	(4.615)	(2.039)	(4.865)
Year Effects	Yes	Yes	Yes	Yes	Yes
Constant	63.54	233.0	-29.95	-4.730	226.9
	(155.0)	(327.6)	(190.4)	(71.68)	(251.6)
Observations	2933	940	1993	1256	1677
sigma_e	578.0	647.1	540.6	235.7	664.5
sigma_u	940.2	1252.2	733.1	284.4	1049.0
rho	0.726	0.789	0.648	0.593	0.714
r2_w	0.484	0.428	0.525	0.477	0.583
r2_b	0.615	0.632	0.673	0.466	0.478
r2_o	0.498	0.426	0.575	0.401	0.452
	**Panel C-Dependent Variable: Average Number of Citations per Publication**				
L.Federally Funded R&D	0.0000568	-0.0000453	0.000113**	0.000218*	0.0000349
	(0.0000414)	(0.0000805)	(0.0000428)	(0.0000821)	(0.0000429)
L.Non-Federally Funded R&D	0.0000606	0.0000684	0.0000329	-0.00000232	0.000102
	(0.0000476)	(0.0000958)	(0.0000492)	(0.0000577)	(0.0000598)
L.Full Time Faculty	-0.00178	-0.0287*	0.0134	0.0184	-0.0124
	(0.0119)	(0.0112)	(0.0114)	(0.0133)	(0.0115)
Year Effects	Yes	Yes	Yes	Yes	Yes
Constant	6.929***	9.662***	5.588***	4.355***	8.741***
	(0.477)	(0.836)	(0.516)	(0.475)	(0.631)
Observations	2933	940	1993	1256	1677
sigma_e	2.168	2.620	1.906	2.396	1.953
sigma_u	2.956	3.950	2.052	2.269	2.732
rho	0.650	0.694	0.537	0.473	0.662
r2_w	0.241	0.167	0.317	0.269	0.245
r2_b	0.188	0.226	0.383	0.130	0.00450
r2_o	0.154	0.00721	0.311	0.200	0.0968

Standard errors in parentheses.

* p<0.05,

** p<0.01,

*** p<0.001.

Because all of the explanatory variables used in the regressions reported in Tables 2 and 3 are lagged relative to the dependent variable, they are temporally exogenous. These regressions confirm that there is a positive and relatively strong association between both the quantity of federal and non-federal R&D funding an institution receives and the subsequent production of chemical knowledge. Moreover, the relative similarity of the effect sizes across different categories of universities suggests that there is no apparent inefficiency in the distribution of funding between public and private or between more and less research-intensive institutions. These results should be reassuring to funders supporting chemical research as they indicate an effective allocation of funds.

**Fig 6:**
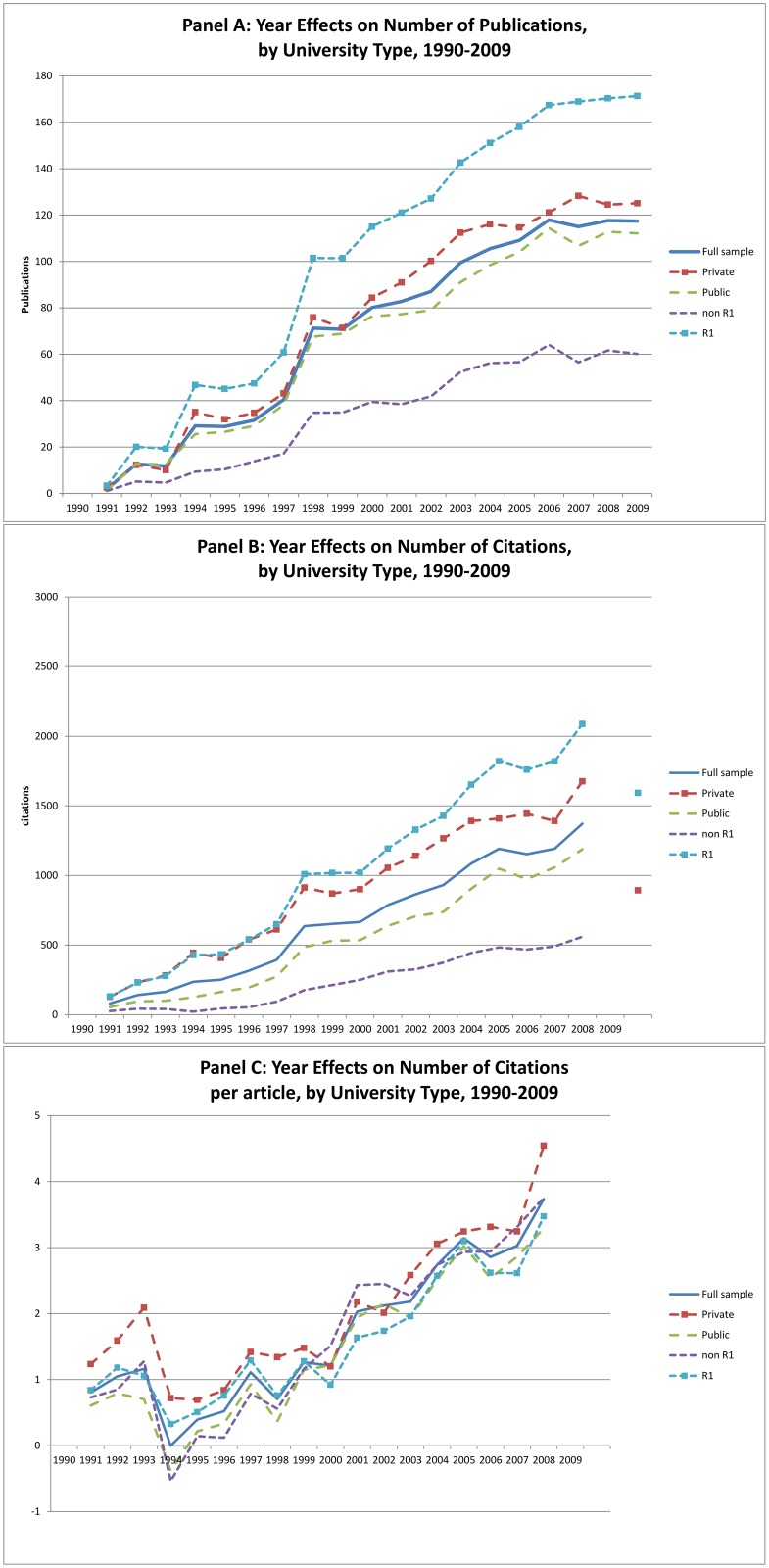
Year Effects from OLS Regression: Number of Publications, Number of Citations and Number of Citations per Article.

The regression results provide further insight into the sources of the rise in publication and citation numbers we described earlier. The year effects (A_t_) included in the regressions capture temporal changes in knowledge output controlling for faculty and funding inputs. In Fig 6 we have plotted year effects for each subset of universities, along with the results for the full sample. These year effects reflect systematic changes in the relationship between inputs and outputs over time, and imply that controlling for funding and faculty numbers academic chemistry experienced a significant positive productivity shock starting in the 1990s. The rising numbers of publications and citations to those publications was common across all of the different categories of universities we consider. However, the number of publications produced and the number of citations to those publications grew considerably more at the Research I universities than the non-Research I universities. Trends in the number of publications were quite similar between public and private universities, but the number of citations received grew more rapidly at the private universities than at the public ones. The difference was not enough, however, to cause any meaningful variation in the time trends of average citations per publication.

#### Instrumental Variable Estimation

The OLS results are not sufficient to fully identify the causal effects of funding on knowledge production. While it is not possible for future publications to directly cause past funding, these two variables may be related through a third, and unobserved variable. In particular we know that funding is allocated purposefully. Thus, investigators with more promising research programs may be more likely to be successful both in seeking funding and in publishing their research results. If this is the case, then the positive correlations revealed by the OLS regressions could simply reflect this unobserved variation in investigator quality, which is positively selected by universities in our sample.

Put somewhat differently, the OLS results are not sufficient to answer the policy-relevant question: how would the quantity of knowledge produced change if an additional $1 million was allocated to chemistry research? Ideally, to answer this question we would want an experimental setting in which additional research funds could be randomly assigned to some universities and not to others. By comparing the impact of funds on the treatment and control groups we could identify the impact of additional research funding on scientific output. We cannot conduct such an experiment, but we can use instrumental variables to obtain estimates of the effects of such truly random variations in funding levels

To measure the true causal effect of funding on research outputs we need to identify the exogenous portion of variations in funding over time and across institutions. Instrumental variable (IV) estimation accomplishes this through the use of one or more instruments (Z) that affect the potentially endogenous explanatory variables (X—in this case funding), but are not otherwise related to the dependent variable (Y—publications and citations). Put somewhat differently, the requirement for a good instrumental variable is, thus that it be correlated with the explanatory variable of interest (funding), but uncorrelated with the error term in the equation. IV estimation is effectively equivalent to estimating a two-stage model: in the first stage the funding variables, X, are modeled as a function of the instruments, Z, and other exogenous variables, while in the second stage the relationship of interest is estimated substituting the predicted value of funding from the first stage.

There are a number of candidate instruments available that we can employ. After exploring these candidates we settled on three: federally funded R&D expenditures for math and physics research, non-federally funded R&D expenditures for math and physics research, and Fall student enrollment. The first two of these instruments should capture important variations in the larger funding environment as well as more institution specific effects such as institutional efforts to encourage increases in sponsored research activity. The last instrument is included to capture effects of tuition revenue as a source expanded institutional resources. A priori, there is no reason to expect that any of these instruments should be correlated with random shocks in the quality of research funding proposals submitted by chemistry faculty at an institution.

Table 4 reports the first stage regression results from the two stage instrumental variable panel regressions for each of the two endogenous explanatory variables—federally-funded chemistry R&D expenditures, and non-federally-funded chemistry R&D expenditures. Overall, the model appears to do a good job of accounting for variation in the endogenous variables as reflected in the relatively large R-squared values. As we might expect, federally-funded R&D in math and physics is strongly and positively significant in the estimates for federally-funded chemistry R&D, while the non-federal math and physics funding variable enters positively and significantly for non-federally funded chemistry R&D. There is, in addition, a strong positive effect of enrollment in both equations, while each of the math and physics expenditure variables exerts a strong positive effect in the regression for the corresponding chemistry variable. The F statistics in Table 4 indicate that we do not have a problem with weak instruments.

**Table 4:** First Stage IV Regressions Determinants of Number of Publications.

	Endogenous Regressor	
	Federally Financed Chemistry R&D Expenditures	Non-Federally Financed Chemistry R&D Expenditures
L.Deflated federally financed R&D expenditures Math & Physics	0.0472*	-0.0028
	(0.02)	(0.008)
L.Deflated non-federally financed R&D expenditures Math & Physics	0.051	0.37***
	(0.027)	(0.0307)
L.Full Time Faculty	-1.971	11.307
	(6.363)	(6.245)
L.Fall Enrollment	0.178***	0.058**
	(0.025)	(0.019)
Year effects	Yes	Yes
Constant	915.25	197.97
	(515.127)	(388.321)
Observations	2933	2933
F-stat (p-value)	38.07(0.000)	41.96(0.000)
sigma_e	1937.38	1460.47
sigma_u	4045.66	2042.82
rho	0.813	0.662
r2_w	0.2407	0.2589
r2_b	0.295	0.5267
r2_o	0.2848	0.4577

Standard errors in parentheses.

* p<0.05,

** p<0.01,

*** p<0.001

Note: Estimates derived from first stage of STATA XTIVREG2 estimation with cluster robust standard errors.

In Table 5 we report the second stage coefficient estimates for the knowledge production function for the full sample and for each subset of institutions. Given the poor fit of the OLS regressions for the average number of citations per article, we have dropped this variable from the IV analysis and focus only on publications and citations received. For the full sample, we find that the effect of federally funded R&D expenditures has increased by a factor of approximately 3 for publications and 3.5 for citations. On the other hand, the effect of non-federally funded R&D expenditures has fallen substantially in magnitude and is no longer statistically significant for either publications or citations. Because our model includes more instruments than potentially endogenous variables the Sargan-Hansen statistic provides an additional confirmation of our a priori interpretation of the instruments. The value of the test statistic indicates that we cannot reject the null hypothesis that regression residuals are uncorrelated with the set of exogenous explanatory variables, indicating that our model is correctly specified.

**Table 5:** IV Regressions. Determinants of Publications, Citations, by University Type. Instruments: Math & Physics Federal R&D, Math and Physics non-federal R&D, Fall Enrollment.

	Full sample	Private	Public	non-Research I	Research l
**Panel A-Dependent Variable: Number of Publications**					
L.Federally Funded R&D	0.0190***	0.0587*	0.0223***	0.00884**	0.00510
	(0.00422)	(0.0258)	(0.00466)	(0.00298)	(0.00312)
L.Non-Federally Funded R&D	0.00327	-0.0510	0.00781**	-0.00632	0.00348
	(0.00247)	(0.0272)	(0.00258)	(0.00340)	(0.00218)
L.Full Time Faculty	-0.181	-0.463	-0.315	0.342*	-0.208
	(0.152)	(0.532)	(0.231)	(0.161)	(0.173)
Constant	-7.493	-148.3	-15.78	14.78*	88.22***
	(13.83)	(131.8)	(13.85)	(6.444)	(18.58)
Observations	2933	940	1993	1256	1677
Sargan-Hansen statistics (p-value)	0.675(0.4114)	0.161(0.6882)	0.085(0.771)	30.015(0.000)	0.927(0.3357)
sigma_e	49.82	116.7	52.86	25.70	47.40
sigma_u	63.76	196.9	59.25	42.62	97.35
rho	0.621	0.740	0.557	0.733	0.808
r2_w	0.497	.	0.449	0.488	0.709
r2_b	0.763	0.749	0.790	0.203	0.685
r2_o	0.693	0.612	0.712	0.295	0.511
**Panel B-Dependent Variable: Number of Citations**					
L.Federally Funded R&D	0.260***	0.700*	0.312***	0.150***	0.132**
	(0.0565)	(0.330)	(0.0562)	(0.0403)	(0.0410)
L.Non-Federally Funded R&D	0.00614	-0.747	0.0703*	-0.0700	0.00460
	(0.0358)	(0.387)	(0.0355)	(0.0397)	(0.0338)
L.Full Time Faculty	-0.620	-7.373	-1.167	3.924	-0.661
	(2.233)	(7.181)	(3.400)	(2.046)	(2.500)
Constant	-740.0***	-1791.8	-999.9***	-179.1*	-107.7
	(190.7)	(1731.2)	(184.9)	(71.94)	(269.5)
Observations	2933	940	1993	1256	1677
Sargan-Hansen statistics (p-value)	0.009(0.924)	0.008(0.9295)	0.731(0.393)	19.157(0.000)	1.685(0.194)
sigma_e	686.7	1532.5	705.7	286.9	687.7
sigma_u	674.3	2327.9	705.1	272.8	854.1
rho	0.491	0.698	0.500	0.475	0.607
r2_w	0.271	.	0.191	0.226	0.554
r2_b	0.730	0.658	0.778	0.380	0.633
r2_o	0.621	0.502	0.645	0.350	0.572

Standard errors in parentheses.

* p<0.05,

** p<0.01,

*** p<0.001.

Results are broadly consistent across the different categories of institutions we have identified, although coefficient magnitudes vary. When we split the sample between private and public institutions, the effect of federally-funded R&D expenditures is larger for private universities (for both publications and citations), and we find that public universities’ non-federally funded R&D expenditures do have a positive and significant, though significantly weaker, effect on research outputs. Given the low level of non-federal R&D expenditures at private universities, it is hardly surprising to find that this category of research funding has no effect for them. When the sample is split between Research I and non-Research I universities, the effect of federally-funded R&D expenditures is much weaker for both subsets of universities than it is for the whole sample and is statistically significant only for the non-Research I universities. This suggests that much of the effect observed in the full sample is coming from variations in the level of federally funded R&D expenditures and research outputs between the two groups, rather than within either group.

**Fig 7:**
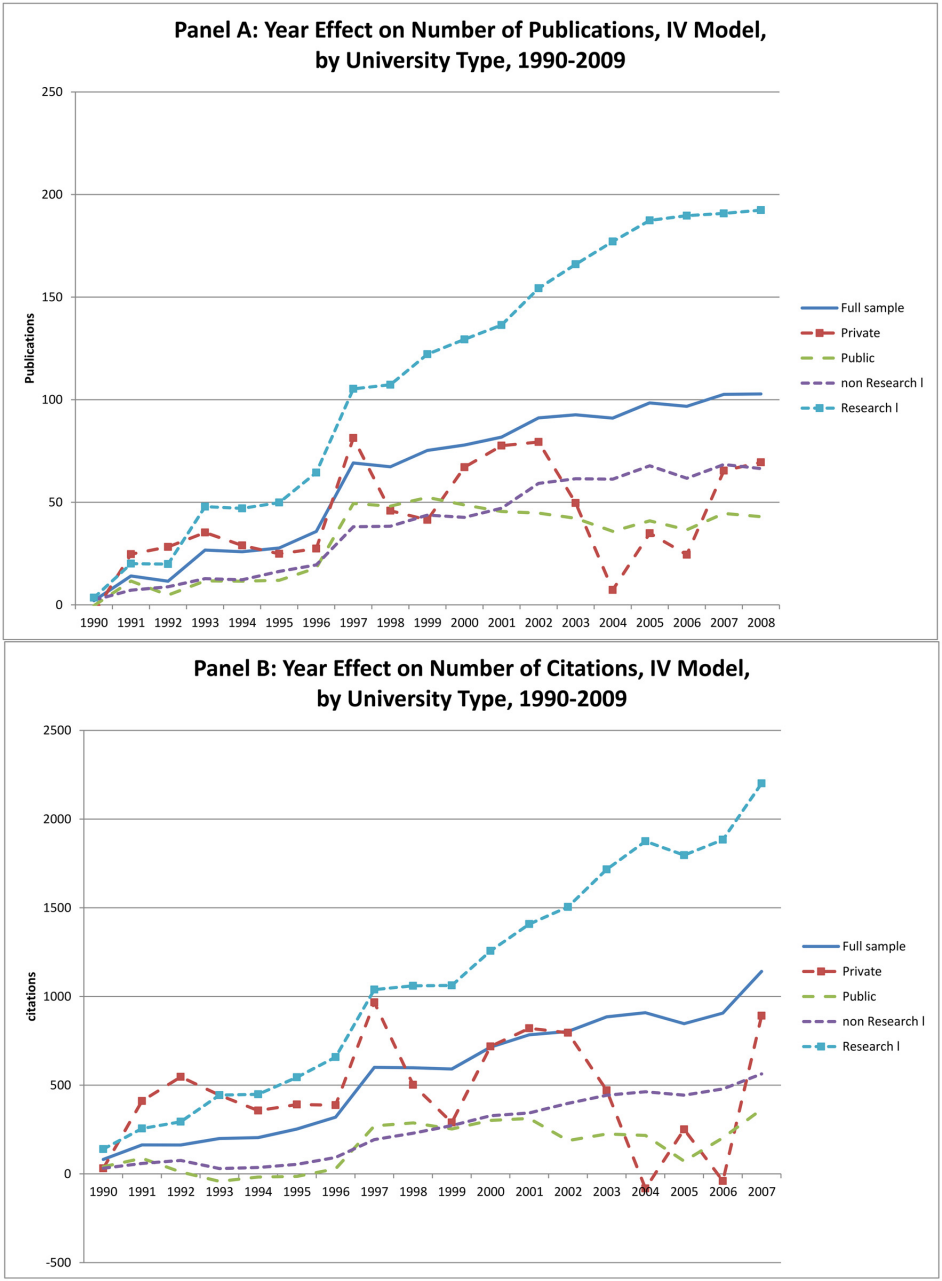
Year Effects from IV Regressions on Number of Publications and Citations.

The year effects, which measure the impact of technological change from the instrumental variable regressions, are summarized in Fig 7. Once again we see that there remains a significant unexplained positive time trend for both publications and citations. This growth is most pronounced for the Research I universities, and weakest for the non-Research I institutions. As was true in the OLS regressions, the trend growth was also larger for private universities than for the publics.

## Discussion

Two results emerge from the regressions described in this section. The first is confirmation of a positive relationship between research funding and knowledge production, whether measured by raw publication numbers or weighting publications by the number of citations that they receive. Universities that receive more funding at the margin, produce more articles and receive more citations to those articles. While the OLS results cannot demonstrate a causal effect of funding on knowledge production, they do confirm that the purposeful allocation of funding across institutions does correspond to the presence of more productive investigators who make collectively larger contributions to chemical knowledge. The IV estimates suggest that there is a causal effect of funding on knowledge production, at least for federal funding. If true, this implies that the marginal effect of increased funding would be relatively large. Using the coefficients from the full sample, they imply that an additional $1 million would lead to the publication of 19 more articles and an additional 260 citations to those articles. This places the marginal cost of an additional article (a bit more than $50,000) roughly in line with the average cost per article across all institutions.

The second striking result to emerge from our analysis is the rapid growth in knowledge production in chemistry over the 20 years from 1990 through 2009. At the moment, this increase appears in our analysis as a residual time effect. In other words, it is unexplained by any of the measurable input variables, but can be considered a proxy for technological change [13]. Compared to the analysis in Adams and Griliches [5] for the 1980s, this appears to be a departure from past experience. Given the coincidence of its timing with the spread of automatic laboratory data collection and analysis using personal computers and the internet, it is possible that the growth in the number of articles published relative to measured inputs reflects an IT mediated increase in the efficiency of academic chemistry. Alternatively, given the greater emphasis on publications and bibliometric measures of influence, it is possible that rising numbers of publications and citations to those publications reflect changes in the ethos of academic chemistry that have reduced the knowledge content of individual publications. Lacking a direct measure of the knowledge contributions of individual articles, we cannot at present distinguish between these alternative mechanisms or apportion the sources of the increase between them. However, preliminary analysis of data on chemistry-related patents issued to the universities in our sample suggests an upward trend in patenting that is consistent with the interpretation of an increase in the productivity of academic chemists.

## Conclusion

In light of the substantial investments made by both federal and non-federal sources in supporting university R&D activities, it is quite reasonable to wonder whether these investments are productive. Only a few previous studies have sought to elucidate these relationships. Several have focused on the level of individual investigators and grants, a perspective that we believe neglects significant spillover effects across researchers. As we have argued, we believe that it is most appropriate to consider relationships at the level of the discipline and to concentrate on aggregate and institution-level variations.

As we have shown here, for the case of academic chemistry, analysis at this level yields important insights about knowledge production and its relationship to sponsored research funding from both federal and non-federal sources. While we have documented the fact that funding goes to those institutions that are most productive, and that there appears to be a causal effect of funding on subsequent knowledge production, we have also documented an intriguing increase in scholarly output among academic chemists in the years we have selected for study. This suggests that technological change may have shifted the production function, increasing the federal government’s return on investment. Resolving the sources of this increase will require further effort to refine measurements of the knowledge embodied in academic publications.

## Supporting Information

S1 Table Annual Average Values of R&D Expenditures and Other Characteristics of Sample Institutions, by Total Federally Funded R&D Expenditures, 1990–2009. (DOCX)

S1 Text Data Appendix. The analysis in this paper rests on merging several different sources of data. We describe the sources and key characteristics of each briefly.
(DOCX)
