# Supplementary material for: The Effects of Research & Development Funding on Scientific Productivity: Academic Chemistry, 1990-2009
Source: PLoS One. 2015 Sep 15;10(9):e0138176. doi: 10.1371/journal.pone.0138176 (PMC4570780; doi:10.1371/journal.pone.0138176)
Supplement: S1 Table — (DOCX) [file pone.0138176.s001.docx]

| **Table S1: Annual Average Values of R&D Expenditures and Other Characteristics of Sample Institutions, by Total Federally Funded R&D Expenditures, 1990-2009** | | | | | | | |
| --- | --- | --- | --- | --- | --- | --- | --- |
|  | R&D Expenditures by Source ($1,000s) | | |  |  |  |  |
| University | Federal | Non-Federal | Total | Ph.D.s | Postdoc Researchers | Publications | Citations over 3 years |
| Massachusetts Institute of Technology | $28,262.9 | $8,574.4 | $36,837.3 | 66 | 141 | 522 | 5,126 |
| California Institute of Technology | $21,095.5 | $4,501.9 | $25,597.4 | 35 | 103 | 301 | 2,830 |
| Johns Hopkins University | $18,735.2 | $1,273.9 | $20,009.1 | 17 | 32 | 253 | 2,496 |
| University of California-Berkeley | $18,705.6 | $8,385.2 | $27,090.8 | 76 | 166 | 632 | 5,681 |
| Stanford University | $18,457.3 | $4,082.3 | $22,539.6 | 44 | 84 | 364 | 3,663 |
| Harvard University | $17,334.5 | $2,121.1 | $19,455.6 | 27 | 129 | 509 | 7,795 |
| Pennsylvania State U, All Campuses | $16,183.6 | $11,788.7 | $27,972.2 | 44 | 58 | 446 | 2,652 |
| University of Illinois at Urbana-Champaign | $15,787.8 | $8,835.3 | $24,623.1 | 52 | 40 | 424 | 3,216 |
| University of Texas at Austin | $15,527.6 | $14,590.3 | $30,117.9 | 64 | 91 | 349 | 2,714 |
| University of California-Los Angeles | $15,171.2 | $4,575.5 | $19,746.7 | 35 | 85 | 330 | 3,439 |
| University of Colorado, All Campuses | $14,894.9 | $4,523.5 | $19,418.4 | 29 | 83 | 256 | 1,855 |
| University of Minnesota, All Campuses | $14,398.6 | $6,406.9 | $20,805.5 | 55 | 75 | 472 | 3,429 |
| Cornell University, All Campuses | $13,828.9 | $6,030.0 | $19,858.9 | 35 | 55 | 357 | 3,093 |
| University of Wisconsin-Madison | $13,779.3 | $8,679.1 | $22,458.4 | 56 | 45 | 426 | 3,101 |
| University of Pennsylvania | $13,773.4 | $2,316.6 | $16,090.0 | 30 | 81 | 342 | 3,321 |
| University of California-San Diego | $12,701.0 | $2,962.1 | $15,663.1 | 21 | 64 | 310 | 3,213 |
| Northwestern Univ | $12,514.9 | $4,934.9 | $17,449.8 | 38 | 84 | 377 | 3,846 |
| Rutgers the State Univ of NJ, All Campuses | $12,373.2 | $5,572.0 | $17,945.2 | 27 | 43 | 256 | 1,431 |
| University of Washington - Seattle | $12,326.7 | $4,411.5 | $16,738.2 | 37 | 47 | 330 | 3,625 |
| Purdue University, All Campuses | $12,134.1 | $8,695.4 | $20,829.5 | 67 | 45 | 401 | 2,535 |
| University of Michigan, All Campuses | $11,832.4 | $5,095.9 | $16,928.2 | 48 | 47 | 452 | 4,162 |
| Georgia Institute of Technology, All Campuses | $11,249.9 | $11,690.5 | $22,940.3 | 35 | 28 | 257 | 2,321 |
| University of Utah | $11,060.0 | $4,505.2 | $15,565.1 | 30 | 52 | 297 | 2,315 |
| University of Pittsburgh, All Campuses | $10,519.2 | $2,503.7 | $13,022.9 | 30 | 59 | 254 | 2,074 |
| University of North Carolina at Chapel Hill | $10,319.3 | $2,725.4 | $13,044.7 | 35 | 57 | 328 | 3,039 |
| Texas A&M University, All Campuses | $10,292.9 | $18,344.9 | $28,637.8 | 58 | 84 | 403 | 2,632 |
| Ohio State University, All Campuses | $10,044.5 | $9,894.5 | $19,939.0 | 40 | 57 | 317 | 1,838 |
| Princeton University | $9,976.0 | $6,087.2 | $16,063.3 | 28 | 62 | 181 | 1,206 |
| University of Notre Dame | $9,974.5 | $1,994.2 | $11,968.7 | 17 | 44 | 177 | 1,316 |
| University of Massachusetts at Amherst | $9,542.5 | $8,312.0 | $17,854.5 | 35 | 35 | 237 | 1,434 |
| Arizona State University Main | $9,279.7 | $6,821.6 | $16,101.3 | 14 | 30 | 133 | 1,095 |
| University of California-Irvine | $9,191.4 | $3,516.3 | $12,707.7 | 25 | 46 | 211 | 1,671 |
| Columbia University in the City of New York | $8,969.1 | $2,676.5 | $11,645.6 | 25 | 59 | 259 | 2,501 |
| University of California-Santa Barbara | $8,871.8 | $3,185.1 | $12,056.9 | 23 | 58 | 241 | 2,406 |
| University of Arizona | $8,857.6 | $4,843.1 | $13,700.7 | 24 | 48 | 242 | 1,306 |
| University of Florida | $8,704.1 | $6,188.6 | $14,892.7 | 46 | 59 | 371 | 2,134 |
| University of Delaware | $8,665.0 | $5,124.4 | $13,789.4 | 27 | 39 | 256 | 1,388 |
| University of South Carolina, All Campuses | $8,569.4 | $6,292.3 | $14,861.6 | 21 | 30 | 122 | 908 |
| Yale University | $8,560.5 | $2,002.2 | $10,562.6 | 22 | 38 | 236 | 2,711 |
| North Carolina State University at Raleigh | $8,496.7 | $11,125.6 | $19,622.3 | 26 | 47 | 264 | 1,382 |
| University of Chicago | $8,460.5 | $2,389.5 | $10,850.0 | 21 | 25 | 185 | 1,650 |
| University of California-Davis | $8,071.5 | $3,523.2 | $11,594.7 | 31 | 45 | 335 | 2,098 |
| Michigan State University | $7,530.1 | $8,531.5 | $16,061.6 | 32 | 46 | 247 | 1,361 |
| University of Virginia, All Campuses | $7,419.8 | $1,689.0 | $9,108.8 | 20 | 40 | 181 | 1,531 |
| Case Western Reserve University | $7,389.1 | $4,425.7 | $11,814.8 | 26 | 15 | 195 | 1,616 |
| Indiana University, All Campuses | $7,274.4 | $6,195.9 | $13,470.3 | 20 | 44 | 216 | 1,631 |
| University of Tennessee Univ-Wide Adm Cent Off | $7,188.0 | $3,434.5 | $10,622.4 | 19 | 25 | 234 | 1,320 |
| University of Maryland at College Park | $7,179.9 | $5,100.2 | $12,280.1 | 21 | 18 | 186 | 1,075 |
| New Mexico State University, All Campuses | $7,061.8 | $1,118.6 | $8,180.4 | 6 | 4 | 50 | 362 |
| Louisiana State Univ, All Campuses | $6,999.2 | $7,160.4 | $14,159.6 | 22 | 31 | 176 | 889 |
| Colorado State University | $6,974.7 | $2,495.8 | $9,470.5 | 17 | 53 | 144 | 1,038 |
| University of Southern California | $6,956.0 | $3,270.7 | $10,226.7 | 22 | 51 | 196 | 1,431 |
| SUNY at Buffalo, All Campuses | $6,952.3 | $5,981.4 | $12,933.7 | 27 | 45 | 182 | 1,184 |
| Virginia Polytechnic Institute and State Univ | $5,994.4 | $7,191.8 | $13,186.1 | 24 | 27 | 204 | 899 |
| Carnegie Mellon University | $5,893.6 | $1,875.6 | $7,769.2 | 21 | 28 | 160 | 1,237 |
| University of Rochester | $5,871.4 | $942.1 | $6,813.6 | 17 | 31 | 145 | 1,242 |
| Rice University | $5,796.1 | $2,454.0 | $8,250.0 | 20 | 32 | 145 | 1,437 |
| Emory University | $5,690.6 | $2,680.7 | $8,371.2 | 16 | 39 | 193 | 1,741 |
| SUNY at Stony Brook, All Campuses | $5,449.7 | $3,986.2 | $9,435.9 | 16 | 19 | 198 | 1,422 |
| University of Southern Mississippi | $5,425.5 | $1,365.7 | $6,791.1 | 11 | 17 | 91 | 276 |
| University of Oklahoma, All Campuses | $5,292.0 | $9,009.5 | $14,301.4 | 15 | 29 | 120 | 670 |
| Rensselaer Polytechnic Institute | $5,024.4 | $2,522.7 | $7,547.1 | 19 | 22 | 117 | 553 |
| Washington University | $4,978.8 | $2,397.4 | $7,376.2 | 18 | 28 | 230 | 2,577 |
| University of Kansas, All Campuses | $4,898.4 | $4,978.9 | $9,877.3 | 22 | 29 | 162 | 1,060 |
| University of Nebraska Central Admin Sys Off | $4,779.8 | $3,782.4 | $8,562.2 | 12 | 17 | 149 | 846 |
| University of Houston | $4,707.2 | $5,677.1 | $10,384.3 | 22 | 30 | 156 | 917 |
| Vanderbilt University | $4,677.5 | $927.0 | $5,604.5 | 11 | 20 | 200 | 2,046 |
| Wayne State University | $4,665.9 | $4,238.4 | $8,904.2 | 22 | 21 | 153 | 1,107 |
| Clemson University | $4,541.9 | $5,466.3 | $10,008.2 | 14 | 12 | 147 | 695 |
| University of Alabama in Huntsville | $4,192.9 | $1,665.8 | $5,858.7 | 0 | 1 | 27 | 81 |
| Iowa State University | $4,150.9 | $3,794.8 | $7,945.7 | 35 | 22 | 335 | 1,906 |
| University of California-Santa Cruz | $4,117.7 | $1,602.1 | $5,719.8 | 8 | 21 | 72 | 476 |
| University of Oregon | $4,069.5 | $1,291.8 | $5,361.3 | 7 | 25 | 74 | 427 |
| University of Illinois at Chicago | $3,983.0 | $2,412.3 | $6,395.4 | 22 | 15 | 187 | 1,346 |
| University of Iowa | $3,951.2 | $2,708.4 | $6,659.6 | 19 | 21 | 171 | 1,340 |
| Montana State University - Bozeman | $3,931.4 | $1,260.0 | $5,191.4 | 6 | 17 | 50 | 274 |
| University of New Mexico, All Campuses | $3,910.9 | $1,534.7 | $5,445.7 | 11 | 15 | 115 | 768 |
| University of California-Riverside | $3,896.9 | $1,568.0 | $5,464.9 | 11 | 34 | 156 | 1,221 |
| Boston College | $3,887.5 | $1,219.3 | $5,106.8 | 9 | 19 | 63 | 611 |
| Florida State University | $3,886.7 | $5,761.0 | $9,647.6 | 11 | 42 | 134 | 730 |
| University of PR Rio Piedras Campus | $3,799.3 | $386.2 | $4,185.6 | 7 | 6 | 37 | 102 |
| Kansas State University | $3,753.1 | $1,303.4 | $5,056.6 | 9 | 14 | 89 | 473 |
| CUNY City College | $3,720.1 | $707.1 | $4,427.2 | 0 | 11 | 37 | 186 |
| Brigham Young University, All Campuses | $3,579.1 | $1,604.5 | $5,183.5 | 10 | 12 | 86 | 390 |
| Mississippi State University | $3,512.1 | $3,248.1 | $6,760.2 | 5 | 10 | 45 | 139 |
| New York University | $3,475.1 | $1,212.0 | $4,687.1 | 8 | 24 | 116 | 1,024 |
| University of Alabama | $3,451.5 | $1,620.2 | $5,071.7 | 10 | 13 | 85 | 476 |
| Duke University | $3,447.2 | $1,864.0 | $5,311.2 | 14 | 32 | 205 | 2,459 |
| University of Akron, All Campuses | $3,446.6 | $8,058.6 | $11,505.2 | 40 | 36 | 138 | 670 |
| University of Dayton | $3,409.9 | $594.0 | $4,004.0 | 0 | 0 | 34 | 132 |
| Washington State University | $3,344.5 | $1,906.0 | $5,250.6 | 7 | 12 | 126 | 665 |
| University of Maryland Baltimore County | $3,326.2 | $789.6 | $4,115.8 | 5 | 13 | 126 | 968 |
| Georgetown University | $3,275.2 | $824.1 | $4,099.4 | 6 | 6 | 88 | 709 |
| Oregon State University | $3,269.4 | $1,196.9 | $4,466.3 | 12 | 12 | 102 | 500 |
| Brown University | $3,219.0 | $2,434.5 | $5,653.5 | 11 | 11 | 82 | 541 |
| University of Arkansas, Main Campus | $3,156.9 | $2,457.6 | $5,614.5 | 8 | 11 | 68 | 371 |
| Northeastern University | $3,131.9 | $1,836.6 | $4,968.5 | 9 | 11 | 80 | 500 |
| University of Kentucky, All Campuses | $3,103.8 | $2,047.1 | $5,150.9 | 14 | 18 | 180 | 1,100 |
| Rockefeller University | $2,961.8 | $2,024.1 | $4,985.9 | 0 | 7 | 44 | 690 |
| Auburn University, All Campuses | $2,959.0 | $3,926.6 | $6,885.6 | 13 | 9 | 84 | 327 |
| University of Tulsa | $2,908.2 | $3,263.4 | $6,171.6 | 7 | 2 | 22 | 16 |
| University of Cincinnati, All Campuses | $2,903.3 | $3,477.4 | $6,380.7 | 21 | 14 | 174 | 1,046 |
| Boston University | $2,878.2 | $346.1 | $3,224.3 | 8 | 6 | 131 | 1,072 |
| New Mexico Institute of Mining and Technology | $2,873.2 | $3,732.8 | $6,606.1 | 3 | 0 | 18 | 72 |
| CUNY Hunter College | $2,839.1 | $1,175.0 | $4,014.1 | 0 | 0 | 41 | 224 |
| Tufts University | $2,838.6 | $934.3 | $3,772.9 | 7 | 14 | 89 | 872 |
| North Dakota State University, All Campuses | $2,815.6 | $1,900.4 | $4,716.0 | 6 | 12 | 87 | 349 |
| Colorado School of Mines | $2,804.7 | $2,410.7 | $5,215.3 | 9 | 7 | 48 | 185 |
| Virginia Commonwealth University | $2,727.7 | $1,659.4 | $4,387.1 | 7 | 8 | 110 | 625 |
| Clark Atlanta University | $2,714.2 | $398.8 | $3,112.9 | 1 | 5 | 18 | 43 |
| Lehigh University | $2,678.7 | $3,227.4 | $5,906.2 | 16 | 19 | 79 | 361 |
| University of Georgia | $2,654.3 | $5,099.0 | $7,753.4 | 17 | 17 | 221 | 1,333 |
| University of Connecticut, All Campuses | $2,532.5 | $2,640.9 | $5,173.4 | 23 | 16 | 178 | 928 |
| Syracuse University, All Campuses | $2,479.3 | $973.5 | $3,452.8 | 10 | 12 | 79 | 410 |
| West Virginia University | $2,440.8 | $1,210.9 | $3,651.7 | 7 | 9 | 67 | 308 |
| Tulane University | $2,384.1 | $1,896.6 | $4,280.6 | 9 | 12 | 88 | 567 |
| Oklahoma State University, All Campuses | $2,350.0 | $2,376.0 | $4,726.0 | 10 | 5 | 74 | 363 |
| Brandeis University | $2,233.7 | $659.7 | $2,893.4 | 7 | 14 | 47 | 394 |
| Jackson State University | $2,206.5 | $327.0 | $2,533.5 | 1 | 9 | 47 | 221 |
| Illinois Institute of Technology | $2,169.6 | $946.5 | $3,116.1 | 9 | 5 | 34 | 125 |
| Clarkson University | $2,144.8 | $2,596.0 | $4,740.8 | 10 | 21 | 57 | 288 |
| New Jersey Institute Technology | $2,136.9 | $2,441.3 | $4,578.2 | 5 | 1 | 36 | 129 |
| Texas Tech University | $2,104.0 | $3,913.5 | $6,017.5 | 11 | 22 | 85 | 433 |
| University of Missouri, Columbia | $2,031.3 | $3,841.1 | $5,872.4 | 13 | 11 | 124 | 568 |
| University of Wyoming | $1,804.3 | $2,737.7 | $4,542.0 | 8 | 12 | 44 | 197 |
| University of Hawaii at Manoa | $1,744.4 | $455.0 | $2,199.4 | 4 | 10 | 72 | 398 |
| Dartmouth College | $1,690.0 | $747.9 | $2,438.0 | 6 | 12 | 65 | 498 |
| Drexel University | $1,653.9 | $953.9 | $2,607.8 | 6 | 6 | 71 | 435 |
| Utah State University | $1,629.6 | $898.0 | $2,527.6 | 3 | 5 | 48 | 283 |
| Norfolk State University | $1,629.3 | $37.6 | $1,666.9 | 0 | 0 | 6 | 4 |
| University of New Hampshire | $1,583.8 | $659.8 | $2,243.6 | 5 | 4 | 40 | 152 |
| San Francisco State University | $1,568.0 | $99.2 | $1,667.2 | 0 | 0 | 18 | 68 |
| Howard University | $1,530.4 | $293.0 | $1,823.4 | 5 | 3 | 57 | 170 |
| University of Denver | $1,510.9 | $283.8 | $1,794.8 | 2 | 5 | 13 | 49 |
| Polytechnic University | $1,491.9 | $1,253.2 | $2,745.2 | 9 | 14 | 43 | 195 |
| California State University-Los Angeles | $1,481.8 | $76.8 | $1,558.6 | 0 | 0 | 29 | 81 |
| University of Idaho | $1,467.7 | $1,414.4 | $2,882.1 | 7 | 9 | 66 | 318 |
| Georgia State University | $1,447.5 | $1,659.7 | $3,107.3 | 3 | 21 | 50 | 263 |
| University of Missouri, Rolla | $1,446.9 | $1,867.3 | $3,314.3 | 10 | 4 | 60 | 232 |
| University of Massachusetts Lowell | $1,379.7 | $1,104.1 | $2,483.8 | 12 | 6 | 59 | 175 |
| University of Louisville | $1,378.5 | $898.8 | $2,277.4 | 7 | 7 | 51 | 369 |
| University of Montana | $1,340.3 | $448.6 | $1,788.9 | 3 | 5 | 21 | 66 |
| University of South Florida | $1,327.3 | $1,743.8 | $3,071.1 | 9 | 10 | 82 | 674 |
| University of PR Mayaguez Campus | $1,300.6 | $680.1 | $1,980.6 | 1 | 0 | 25 | 35 |
| North Carolina Agricultural & Tech State Univ | $1,281.5 | $89.5 | $1,371.0 | 0 | 0 | 13 | 24 |
| Stevens Institute of Technology | $1,252.7 | $400.2 | $1,652.9 | 4 | 4 | 27 | 118 |
| Cleveland State University | $1,178.6 | $518.8 | $1,697.4 | 5 | 4 | 24 | 107 |
| Source: See Data Appendix. | | | | | | | |
