# Supplementary material for: The Effects of Research & Development Funding on Scientific Productivity: Academic Chemistry, 1990-2009
Source: PLoS One. 2015 Sep 15;10(9):e0138176. doi: 10.1371/journal.pone.0138176 (PMC4570780; doi:10.1371/journal.pone.0138176)
Supplement: S1 Text — The analysis in this paper rests on merging several different sources of data. We describe the sources and key characteristics of each briefly. (DOCX) [file pone.0138176.s002.docx]

**Data Appendix**

**Research & Development Expenditures**

These data are derived from the National Science Foundation’s Survey of Research and Development Expenditures at Universities and Colleges/Higher Education Research and Development Survey (<http://webcaspar.nsf.gov>). Data are available annually since 1973 for total and federally funded R&D expenditures by discipline. They are obtained from survey responses completed by institutions of higher education, which are responsible for classifying all research expenditures by discipline. We computed non-federally funded R&D expenditures as the difference between total and federally funded R&D expenditures.

Sample institutions were selected from the universe of institutions represented in this data by summing real federally funded R&D expenditures (in prices of 2005) for chemistry and chemical engineering between 1990 and 2009 and then ranking institutions in descending order. We initially selected the top 150 institutions but as described in the text were obliged to drop three of these from the analysis because of inconsistencies in coverage. Before adopting this sampling strategy, we examined several other rankings, using total R&D expenditures and using nominal rather than real expenditures. The lists produced in each case were quite similar. The full list of institutions included in the study in declining order of federally-funded chemistry R&D expenditures is provided as S1 Table.

Institutions report these data for the fiscal year corresponding most closely to the federal fiscal year. In most cases this is likely to run from July of one year to June of the following calendar year. Data are labeled with the calendar year in which the fiscal year ends. Hence data for 2009 most likely cover expenditures from July 2008 through June 2009.

In addition to the expenditures data, this source also contains information on type of control (private or public) and standardized Carnegie Classifications that we use to categorize university types.

**Graduate Students, Doctorates Awarded and Postdoctoral Researchers**

These data are derived from the National Science Foundation and National Institutes of Health Survey of Graduate Students and Postdoctorates in Science and Engineering (graduate student survey) which is conducted annually by the National Center for Science and Engineering Statistics. The survey is conducted in the fall semester of each academic year and data are collected at the department level. These data are available from <http://webcaspar.nsf.gov> . Data are labeled with the calendar year in which they were collected. For example, data labeled 2008 refer to enrollment for the academic year 2008/09, and doctorates awarded are for the 12 months ending in September 2008.

The level of institutional detail provided in this survey is greater than in the R&D expenditure data. In the latter survey a number of multi-campus state systems report a single aggregated number. To link the data sets, we were obliged to aggregate the data in the student survey to match the level of aggregation of the R&D data.

**Faculty**

Faculty counts were hand collected from the American Chemical Society (ACS) faculty directory. Data on full-time staff were collected for departments identified as Chemistry or Chemical Engineering. The directory is published every 2 years, and intervening years were imputed by linear interpolation. The ACS directories are issued in odd numbered years and the faculty counts are for September of the previous year (e.g., the 1991 directory reports faculty in September of 1990). Not every department reported staff numbers in every year, so some additional interpolation was required to fill in additional missing values. In no case were data imputed if data were not available for at least half of the observations possible. As a result of the imputation process, some faculty numbers are expressed as fractions. Some faculty counts are fractional in the original data as well reflecting the presence of faculty with part time appointments converted to Full Time Equivalents.

Faculty counts were available for Chemistry at all institutions, but the chemical engineering data were not always reliably available, so faculty numbers for chemical engineering were included only when they could be reliably estimated for all years considered in the analysis. This avoids the problem of spurious fluctuations created by the entry and exit of units from the data set, but does mean that faculty counts may be too low in a few cases, where data were reported sporadically. The difference in levels will, however, be absorbed in institution level dummy variables.

Data are labeled for the calendar year in which they were collected, thus labeled 2008 (reported in the 2009 ACS directory) would be faculty numbers as of September 2008.

**Publications and Citations**

Publication and citation data were computed by Thomson Reuters, Research Analytics from the data underlying the Web of Science publication and citation database. Thomson Reuters subject area experts categorize journals into subject classes based on detailed analysis of the content and focus of the journals. See http://wokinfo.com/media/essay/journal_selection_essay-en.pdf for additional details regarding the selection process used by Thomson Reuters in compiling the Web of Science data. The Web of Science is relatively selective about which journals are included, reflecting subject expert judgment and objective metrics of journal impact. Our research began with the full set of journals that Thomson Reuters categorizes as Chemistry and Chemical Engineering. We also conducted an analysis of all journal titles indexed by Thomson Reuters and added a small number of additional journals that contain significant chemistry content.

We then worked closely with Thomson Reuters staff to match publications by author affiliation to universities in our sample. In addition to institution name, we considered city, state and zip code information associated with authors to verify the accuracy of article linkages.

After verifying the full list of publications, Thomson Reuters analyzed them to produce summary statistics describing the number of publications each year produced by each institution, the number of citations that those publications received in 3 and 5 year windows beginning with the publication year, and a variety of other citation related metrics.

**Fall Student Enrollment**

These data come from the IPEDS Enrollment Survey and were downloaded from <http://webcaspar.nsf.gov> . The original source is the Higher Education General Information Survey (HEGIS) and the Integrated Postsecondary Education Data System (IPEDS) that is conducted by the Department of Education's National Center for Education Statistics (NCES).

Data are labeled with the calendar year in which they are collected. So enrollment statistics labeled 2008 reflect enrollment during the Fall semester of the 2008/09 Academic Year.
